# Downregulation of endogenous nectin1 in human keratinocytes by herpes simplex virus 1 glycoprotein D excludes superinfection but does not affect NK cell function

**DOI:** 10.1099/jgv.0.001969

**Published:** 2024-03-12

**Authors:** Joanne Kite, Monica Hill, Natasha Preston, Anzelika Rubina, Simon Kollnberger, Eddie Chung Yern Wang, Gillian Elliott

**Affiliations:** 1 Section of Virology, Department of Microbial Sciences, School of Biosciences, University of Surrey, Guildford GU2 7XH, UK; 2 Division of Infection & Immunity, School of Medicine, Cardiff University, Cardiff CF14 4XN, UK

**Keywords:** HSV1, nectin1, keratinocyte, virus receptor, glycoprotein D, NK cell ligand, superinfection exclusion

## Abstract

Many viruses downregulate their cognate receptors, facilitating virus replication and pathogenesis via processes that are not yet fully understood. In the case of herpes simplex virus 1 (HSV1), the receptor binding protein glycoprotein D (gD) has been implicated in downregulation of its receptor nectin1, but current understanding of the process is limited. Some studies suggest that gD on the incoming virion is sufficient to achieve nectin1 downregulation, but the virus-encoded E3 ubiquitin ligase ICP0 has also been implicated. Here we have used the physiologically relevant nTERT human keratinocyte cell type – which we have previously shown to express readily detectable levels of endogenous nectin1 – to conduct a detailed investigation of nectin1 expression during HSV1 infection. In these cells, nectin1, but not nectin2 or the transferrin receptor, disappeared from the cell surface in a process that required virus protein synthesis rather than incoming virus, but did not involve virus-induced host shutoff. Furthermore, gD was not only required but was sufficient for nectin1 depletion, indicating that no other virus proteins are essential. NK cells were shown to be activated in the presence of keratinocytes, a process that was greatly inhibited in cells infected with wild-type virus. However, degranulation of NK cells was also inhibited in ΔgD-infected cells, indicating that blocking of NK cell activation was independent of gD downregulation of nectin1. By contrast, a superinfection time-course revealed that the ability of HSV1 infection to block subsequent infection of a GFP-expressing HSV1 was dependent on gD and occurred in line with the timing of nectin1 downregulation. Thus, the role of gD-dependent nectin1 impairment during HSV infection is important for virus infection, but not immune evasion, which is achieved by other mechanisms.

## Introduction

Several viruses have been shown to downregulate their cognate receptors at various stages after they have entered the cell and initiated intracellular replication, for example: human immunodeficiency virus (HIV) and the CD4 receptor on T cells [1,2]; measles virus and the CD150/SLAM receptor on B cells [3]; and coronaviruses including severe acute respiratory syndrome coronavirus 2 (SARS CoV2) and the Ace2 receptor on epithelial cells [4,5]. In some cases, the virus-encoded receptor binding protein is required for receptor downregulation, such as spike in SARS-CoV2 [4], whereas in others such as HIV, virus-encoded accessory proteins are involved in downregulation [6,7]. In the case of herpes simplex virus type 1 (HSV1), there is some evidence that this virus alters the localization of its major receptor, the cell adhesion molecule nectin1 [8], but existing data are limited and somewhat inconsistent. Early work suggested that nectin1 was reorganized at the cell surface, particularly in cells in contact with uninfected cells, to facilitate virus spread and entry [9,10], but other work suggested that rather than just being reorganized, nectin1 was downregulated [11,12]. Incoming virus was initially proposed to be sufficient for nectin1 relocalization within 5 min of infection [12], but later work appeared to place nectin1 reorganization at a later time in the infectious cycle [13]. There is also evidence that the nectin1 receptor binding protein glycoprotein D (gD) is both necessary and sufficient for nectin1 reorganization [9,10, 12], but one study has also implicated both the viral E3 ubiquitin ligase ICP0 and the cellular E3 ligase Cbl in the process [13].

Although nectin1 is proposed to be downregulated by HSV1 infection, the purpose for this is not yet clear. Recent work has shown that nectin1 can interact with the NK cell receptor CD96 and that overexpression of nectin1 in K562 erythroleukaemia cells increased cellular susceptibility to NK cell cytotoxicity [14], leading to the proposal that nectin1 can act as an activating NK ligand. Downregulation of nectin1 might therefore be an immune-evasion strategy employed by HSV1 to allow infected cells to avoid NK cell killing. Alternatively, since the expression of gD has been shown to block subsequent virus infection [15], the removal of nectin1 from the infected cell surface may be required for superinfection exclusion, a process that is widespread across other virus families [16,19]. Hence, the mechanism and consequences of nectin1 reorganization during HSV1 infection remain to be fully defined.

Crucially, most studies on the behaviour of nectin1 to date have been in cells of unclear relevance for HSV1 infection in humans, and have frequently involved exogenous overexpression of nectin1. In the human host, HSV1 infects keratinocytes in the stratified epithelial cells of the skin, oral mucosa, cornea or the genital mucosa before transmitting to and establishing lifelong latent infection in sensory neurons [20]. We have been studying HSV1 infection in the nTERT keratinocyte line, a diploid line derived from primary human keratinocytes [21], which offers a tractable system that is physiologically relevant for HSV1 infection. Of note, HSV1 enters and replicates rapidly in these human keratinocytes [22]. Although HSV1 has been shown to use a number of different receptors [8,23,26], we have recently demonstrated that nectin1 is the major receptor for HSV1 entry into nTERT keratinocyte cells [22,27]. Importantly, unlike other cell types that have been used in the field, nTERT cells express relatively high levels of nectin1, and endogenous nectin1 is readily detectable at the cell surface by flow cytometry or immunofluorescence [27]. The nTERT keratinocyte system therefore represents a valuable tool for investigating the downstream effect of HSV1 infection on its endogenous receptor in a physiologically relevant cell type.

Here we show that HSV1 specifically downregulates endogenous nectin1 from the cell surface of infected nTERT keratinocytes, the first time this has been investigated in the cell type that HSV1 infects in the human host. Moreover, we demonstrate that newly synthesized gD targeted to the cell surface is both necessary and sufficient for the process. Despite nectin1 being shown previously to act as an NK cell activating ligand, our results show that keratinocyte induction of NK cell activation does not correlate with the level of nectin1 on the cell surface. However, superinfection exclusion was shown to directly correlate with gD-dependent removal of nectin1 from the cell surface, with cells infected with a ΔgD virus where nectin1 is not relocalized remaining susceptible to HSV1 infection. Hence, we propose that the main purpose of gD-dependent nectin1 depletion in its host cell is to block the availability of the main HSV1 receptor in keratinocytes for downstream virus entry.

## Methods

### Cells and viruses

nTERT cells were cultured in 3 : 1 Dulbecco’s modified Eagle media (DMEM) to Hams F12 media supplemented with RM+supplement [10 ng ml^−1^ mouse epidermal growth factor (Serotec), 1 ng ml^−1^ cholera toxin (Sigma) to increase intracellular cAMP levels, 400 ng ml^−1^ hydrocortisone (Sigma) to enhance cellular morphology, 5 µg ml^−1^ apo-transferrin (Sigma) to increase iron uptake and 13 ng ml^−1^ liothyronine (Sigma), 50 U ml^−1^ penicillin streptomycin and 10 % FBS (Invitrogen)] [28]. Vero cells constitutively expressing gD (VD60) have been described before [29]. Vero cells were cultured in DMEM supplemented with 10 % FBS. Viruses were routinely propagated in Vero cells, with titrations carried out in DMEM supplemented with 2 % FBS and 1 % human serum. Wild-type (Wt) HSV1 strains 17 (s17) and Sc16 were used routinely. The s17 virus expressing GFP-tagged VP22 has been described before [30]. A GFP-expressing reporter virus was constructed in Sc16 by homologous recombination of a plasmid expressing GFP driven from the ICP0 promoter flanked with 200 bp of the virus genome sequence on either side of the UL26–UL27 intergenic region. Strain 17 deleted for ICP0 or the vhs (virion host shutoff) gene UL41 has been described before [31,32], and both were kindly provided by Roger Everett (Centre for Virus Research, Glasgow, UK). The ΔgD and ΔgD variant viruses have been described before [29,33] and were kindly provided by Colin Crump (University of Cambridge, UK).

### Antibodies

Antibodies for gD (LP14) VP16 (LP1) and glycoprotein M (gM) were kindly provided by Colin Crump. Other antibodies were purchased commercially: nectin1 and nectin2 (Biolegend); α-tubulin (Sigma); GFP (Clontech); ICP0, ICP27 and CD71 (Santa Cruz); and Cbl (Abcam). IRDye secondary antibodies were from LICOR Biosciences.

### Drug treatments

Drugs were added to cells 1 h before infection as follows: cytosine β-d-arabinofuranoside (100 ng ml^−1^); actinomycin D (5 µM); cycloheximide (100 ng ml^−1^); and a control of DMSO or water only was used alongside.

### Transfections

Small interfering RNAs (siRNAs) to Cbl (Ambion, ThermoFisher Scientific) were reverse transfected with Lipofectamine 2000 to a final concentration of 20 nM and left for 24 h prior to a second round of siRNA forward transfection. Cells were infected after a further 24 h. The Silencer Select negative control siRNA number 1 was used as a negative control (Ambion, ThermoFisher Scientific).

### SDS-PAGE and Western blotting

Protein samples were analysed by SDS-PAGE and transferred to nitrocellulose membrane for Western blot analysis. Western blots were developed using SuperSignal West Pico chemiluminescent substrate followed by exposure to X-ray film, or by imaging on a LICOR Odyssey Imaging system.

### Immunofluorescence

Cells for immunofluorescence were grown on coverslips and fixed with 4 % paraformaldehyde in PBS for 20 min at room temperature. Cell surface staining was carried out on fixed cells, where fixed cells were permeabilized with 0.5 % Triton-X100 for 20 min for total cell staining. Cells were blocked by incubation in PBS with 10 % newborn calf serum for 20 min, before the addition of primary antibody in PBS with 10 % serum, and a further 30 min of incubation. After extensive washing with PBS, the appropriate Alexafluor conjugated secondary antibody was added in PBS with 10 % serum and incubated for a further 20 min. The coverslips were washed extensively in PBS and mounted in Mowiol containing DAPI to stain nuclei. Images were acquired using a Nikon A1 confocal microscope and processed using ImageJ software [34].

### Flow cytometry

Cell surface staining for nectin1 was performed on live cells that had been blocked by incubation in PBS with 5 % FBS and 1 mM EDTA for 10 min. Mouse anti-nectin1 was added in PBS with 5 % FBS and 1 mM EDTA for 30 min. After extensive washing with PBS, Alexafluor 488 anti-mouse secondary antibody was added in PBS with 5 % FBS and 1 mM EDTA and incubated for a further 30 min. Cells were then washed before being fixed in 1 % paraformaldehyde (PFA) for 20 min, suspended in PBS with 1 % FBS and 1 mM EDTA and analysed using a BD FACS Celesta flow cytometer.

### NK cell lines and CD107a degranulation assays

CD14^−^CD3^−^CD56^+^ NK cells were purified directly *ex vivo* via FACS and stimulated to generate NK cell lines as described previously [35]. nTERT cells were infected with HSV1 at an m.o.i. of 5, or mock-infected. Cells were detached 15 h post-infection using TrypLE Express (Thermo Fisher Scientific) and co-cultured with rested NK cell lines in the presence of anti-CD107a antibody (clone H4A3; BioLegend) and BD GolgiStop (BD Biosciences). NK cell degranulation assays were performed as described previously [36], using an effector:target ratio of 10 : 1. Flow cytometric analysis was used to measure responses from CD3^−^CD56^+^ NK cells. Antibodies and reagents used for flow cytometry staining included Live/Dead Aqua (Thermo Fisher Scientific), anti-CD3 (clone HIT31; BioLegend) and anti-CD56 (clone 5.1H11; BioLegend). Cells were fixed with 4 % paraformaldehyde, and analysed on an Attune NxT flow cytometer (Thermo Fisher Scientific). Data were analysed using FlowJo V10 software. Statistical significance was determined using Brown–Forsythe ANOVA with Dunnett’s T3 multiple comparison post-test. NK CD107a degranulation summary data were analysed using a Wilcoxon matched-pairs signed rank test as the data failed Shapiro–Wilk and Kolmogorov–Smirnov normality testing. Statistical analysis was performed using GraphPad Prism software. *P*-values of <0.05 were considered significant.

## Results

### Endogenous nectin1 is specifically downregulated in nTERT human keratinocytes infected with HSV1

**Fig. 1.**
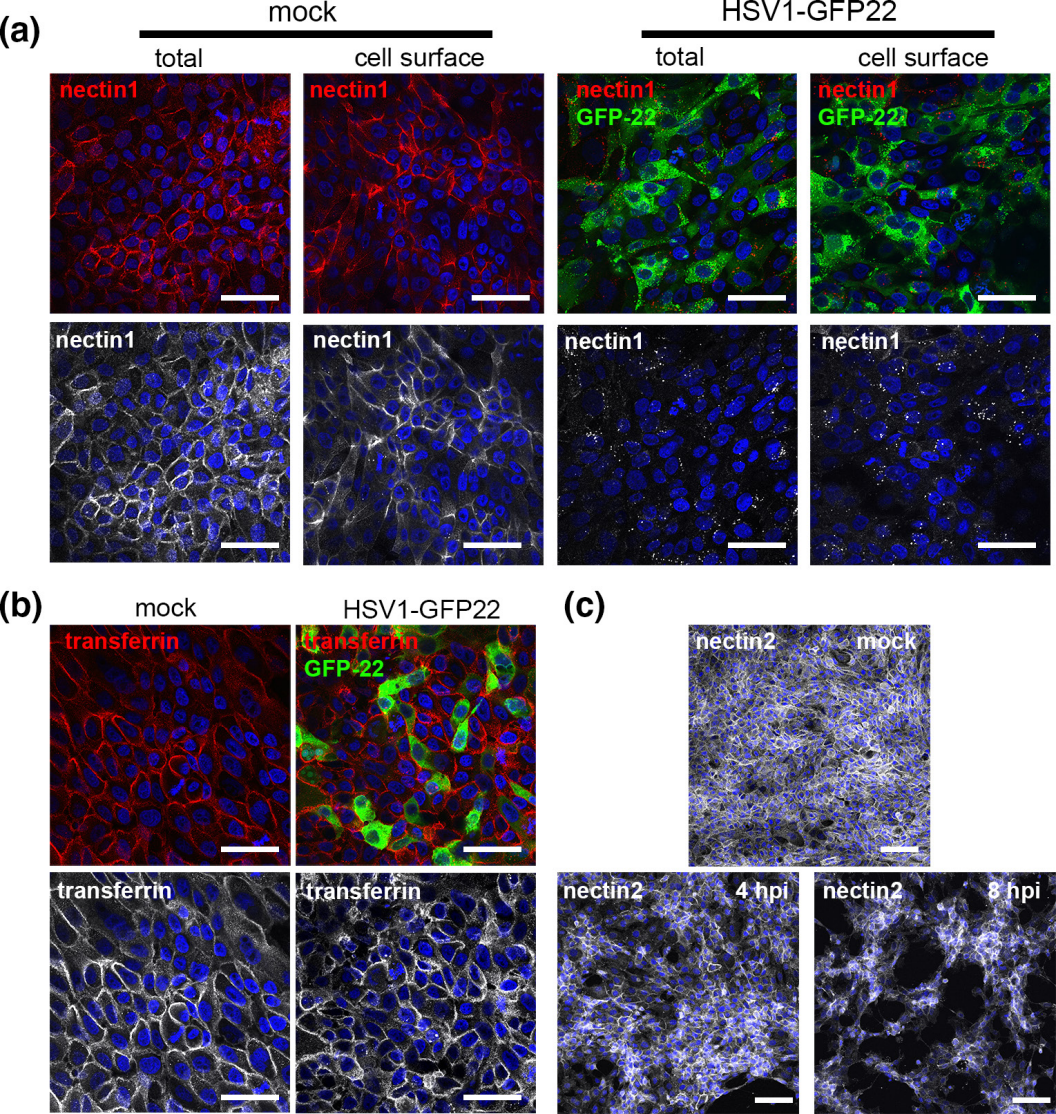
HSV1 infection of human keratinocytes results in downregulation of nectin1 from the cell surface. (**a**) nTERT keratinocytes were left uninfected (mock) or infected with HSV1 (strain 17) expressing GFP-tagged VP22 (HSV1-GFP22). Cells were fixed after 5 h with 4 % paraformaldehyde and either processed straight away for immunofluorescence (cell surface), or permeabilized with 0.5 % Triton-X100 (total). Cells were stained with monoclonal anti-nectin1 (red/white) and imaged for GFP (green) with nuclei stained with DAPI (blue). Bar, 50 µm. (**b**) As for (a), but cells were stained for transferrin receptor (red/white) instead of nectin1. Bar, 50 µm. (**c**) As for (a), but cells were infected with Wt HSV1 and stained for nectin2 (white). Bar, 100 µm.

As a preliminary investigation of endogenous nectin1 in HSV1-infected nTERT keratinocyte cells, we wished to measure the overall level of nectin1 after virus infection. To date we have not found an antibody that detects endogenous nectin1 by Western blotting, even in nTERT cells that express a high level of the protein, but nectin1 is detectable in these cells by immunofluorescence and flow cytometry [27]. We have previously shown that HSV1 enters and replicates rapidly in nTERT keratinocyte cells [22] and hence, to initially determine the effect of virus infection on nectin1 localization, nTERT cells were infected with HSV1 expressing GFP-tagged VP22 [30] at high m.o.i. and fixed after 5 h. Cells were then either processed directly for immunofluorescence to stain only cell surface proteins or were permeabilized and stained to detect intracellular as well as cell surface protein. As previously shown in uninfected cells [27], nectin1 was localized to the cell surface of nTERT keratinocytes and the nectin1 antibody detected the protein at the cell surface, confirming that it recognized the extracellular domain of the protein (Fig. 1a, mock). However, in HSV1-infected cells the characteristic cell surface pattern of nectin1 had disappeared by 5 h and nectin1 was not detectable within the permeabilized and stained cells, suggesting that nectin1 had been depleted during infection (Fig. 1a, HSV1-GFP22). To confirm that this behaviour was specific to nectin1, and not a global effect on cell surface proteins during HSV1 infection, the localization of two additional cell surface proteins was assessed. Cell surface staining of HSV1-GFP22-infected nTERT cells with an antibody for the transferrin receptor revealed that this receptor was present on the cell surface of both mock and infected cells at 5 h, and although the morphology of the cells had slightly changed in response to HSV1 infection, there was little or no downregulation of transferrin receptor (Fig. 1b). In addition, staining of Wt-infected nTERT cells fixed at 4 and 8 h for the highly related protein nectin2, which we have previously shown does not function as an entry receptor for HSV [27], indicated that as for transferrin and in contrast to nectin1, nectin2 remained at the cell surface of HSV1-infected cells (Fig. 1c). This suggests that HSV1 infection specifically targets nectin1 for downregulation from the cell surface.

**Fig. 2.**
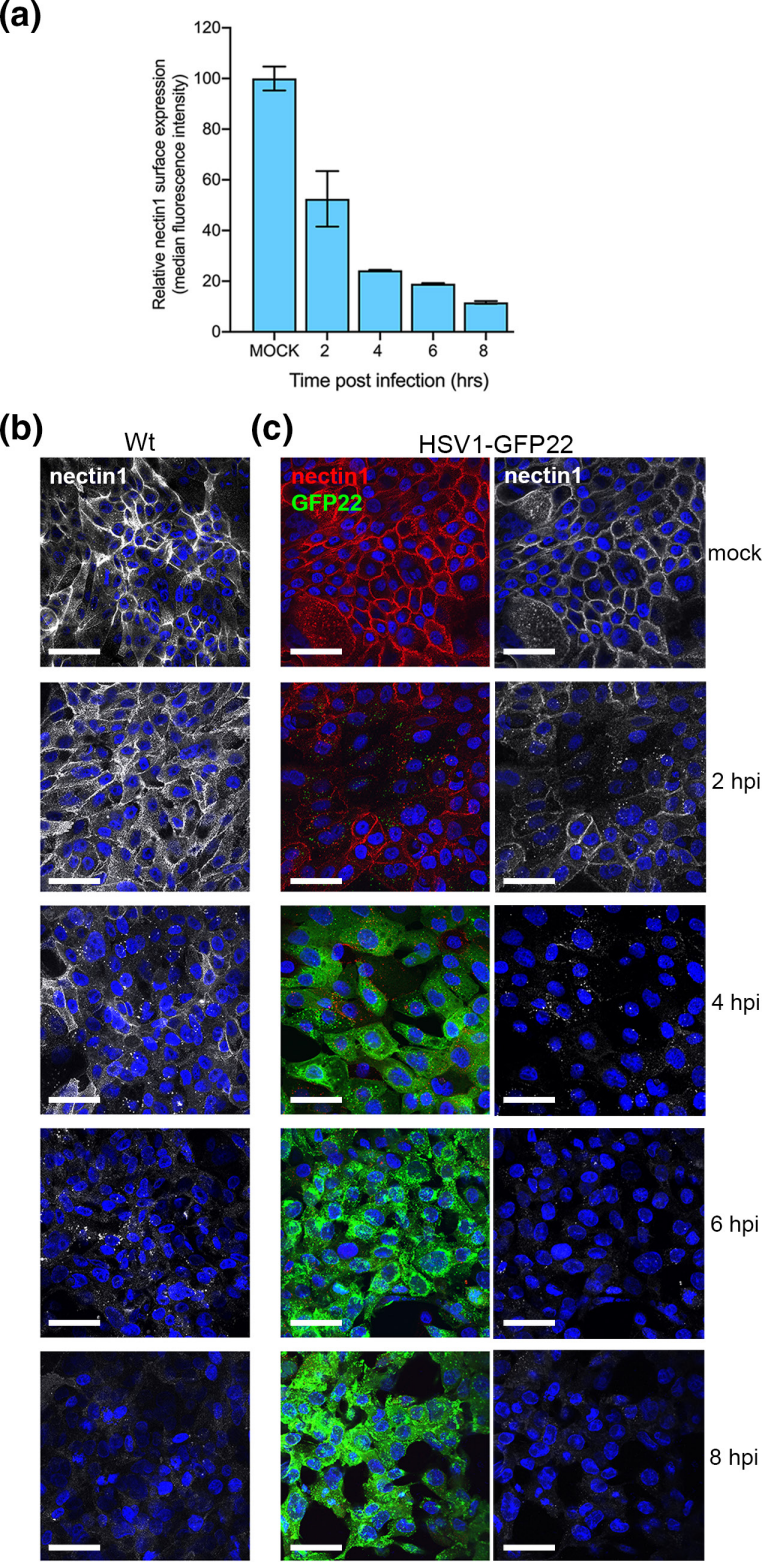
Nectin1 is downregulated in HSV1-infected nTERT keratinocytes early in infection. (**a**) nTERT cells were infected with HSV1 Sc16 at an m.o.i. of 5, harvested at the indicated times and cell-surface stained for nectin1. Nectin1 expression was analysed by flow cytometry and quantified by median fluorescence intensity (mean±sem, *n*=3). (**b**) nTERT cells grown on coverslips were infected with HSV1 Sc16 (Wt) at an m.o.i. of 5 and fixed at the indicated times. Cells were cell-surface stained for nectin1 (white) and nuclei stained with DAPI. Bar, 50 µm. (**c**) As for (**a**) but cells were infected with HSV1 expressing GFP-tagged VP22 (HSV1-GFP22), stained for nectin1 (red/white) and nuclei (blue), and imaged for GFP (green). Bar, 50 µm.

To pinpoint the timing of nectin1 downregulation, nTERT cells were infected with Wt HSV1, fixed at increasing times post-infection and cell-surface stained for nectin1. Quantification of cell surface nectin1 by flow cytometry indicated that the amount of nectin1 at the cell surface was already reduced by 2 h, although the exact amount of nectin1 at this time varied from cell to cell as indicated by large error bars, and continued to reduce until 8 h (Fig. 2a). Imaging of cell surface nectin1 in cells treated in the same way indicated that the protein had relocalized to discrete clusters at the cell surface by 4 h, and by 8 h, nectin1 had disappeared from the cell surface of all cells (Fig. 2b, Wt). Likewise, nTERT cells infected with HSV1-GFP22 exhibited a similar depletion of nectin1 from the cell surface over time (Fig. 2c, HSV1-GFP22).

### Newly synthesized viral proteins are necessary for nectin1 downregulation

**Fig. 3.**
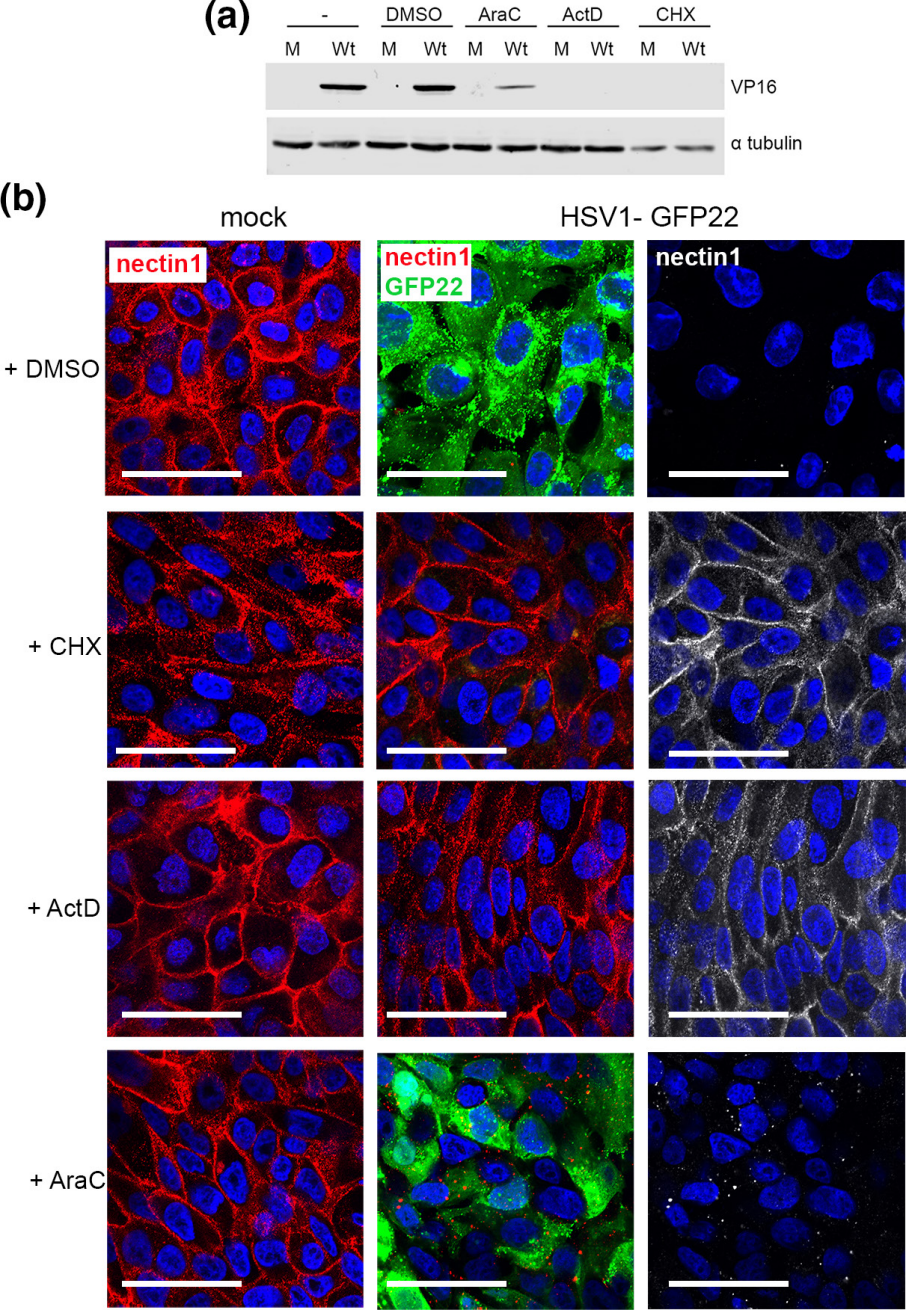
Downregulation of nectin1 in HSV1-infected cells requires *de novo* virus protein synthesis but not genome replication. (**a**) nTERT cells were infected with HSV1 GFP-VP22 at an m.o.i. of 5 in the presence or absence of AraC (100 ng µl^–1^), ActD (5 µM) or CHX (100 ng µl^–1^). Eight hours post-infection lysates were harvested and subjected to SDS-PAGE and Western blotting for VP16 and α-tubulin as a loading control. (**b**) As in (**a**) but nTERT cells grown on coverslips were fixed and cell-surface stained for nectin1 (red/white). Confocal microscopy was used to assess GFP fluorescence (green) and nuclei were stained with DAPI (blue). Bar, 50 µm.

It has been shown previously in experiments on cells overexpressing nectin1 that downregulation occurs within 5 min of virus infection, an activity that has been attributed to incoming virions [11,12]. Given the later timing of endogenous nectin1 downregulation in our human keratinocyte system, we next carried out infection in the presence of several drug inhibitors that act at different stages of HSV1 infection: actinomycin D (ActD) to inhibit transcription, cycloheximide (CHX) to inhibit translation and cytosine arabinoside (AraC) which allows early stages of virus infection to proceed but prevents viral genome replication. Western blotting of inhibitor-treated cells harvested at 8 h indicated that, as expected, expression of the virus protein VP16 was blocked in the presence of ActD or CHX, but not in the presence of AraC, although VP16 was present at a lower level in the AraC-treated cells in line with the inhibition of genome replication (Fig. 3a). nTERT cells infected with HSV1-GFP22 and treated in the same way were fixed at 8 h post-infection (hpi) and cell-surface stained for nectin1. While each of the drug treatments had little effect on nectin1 localization at the cell surface in mock-infected cells (Fig. 3b, mock), nectin1 was downregulated from the cell surface of both untreated and AraC-treated cells, indicating that the effect on nectin1 localization can occur prior to genome replication (Fig. 3b, HSV1-GFP22, +AraC). By contrast, nectin1 remained on the cell surface of ActD- and CHX-treated cells, suggesting that, in contrast to previous studies, new viral protein production is necessary for the downregulation of endogenous nectin1 in nTERT human keratinocytes (Fig. 3b, HSV1-GFP22, +ActD, +CHX). This suggests that entry of the HSV1 virion is not sufficient for nectin1 downregulation in our model system.

### Nectin1 downregulation is not a consequence of HSV1-induced host shutoff

**Fig. 4.**
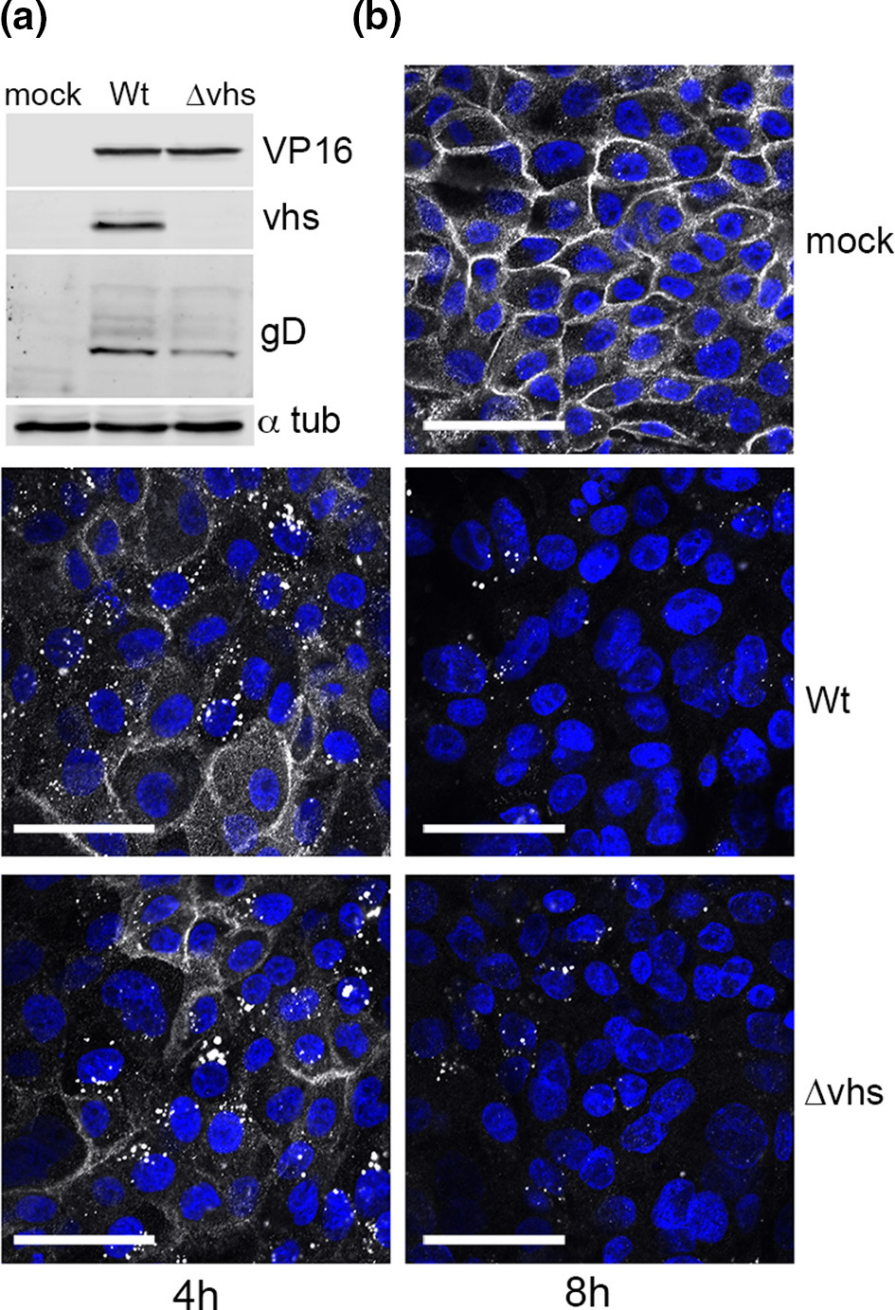
Downregulation of nectin1 in HSV1-infected cells is not a consequence of host shutoff. (**a**) nTERT cells were infected with HSV1 Wt (**s17**) or Δvhs viruses at an m.o.i. of 5. Eight hours post-infection lysates were harvested and subjected to SDS-PAGE and Western blotting for VP16, vhs, gD and α-tubulin as a loading control. (**b**) As in (**a**) but nTERT cells grown on coverslips were fixed at 4 or 8 h and cell-surface stained for nectin1 (white), nuclei were stained with DAPI (blue) and images acquired using confocal microscopy. Bar, 50 µm.

HSV is known to alter the cellular proteome through the action of its endoribonuclease vhs, which targets cellular mRNAs for degradation. The half-life of the nectin1 protein is not known, and as such it is possible that the cell surface loss of nectin1 is a consequence of vhs-induced nectin1 or global mRNA decay. To test this, nTERT cells were infected with either Wt (s17) virus or a Δvhs virus based on s17 [32], then fixed and stained for nectin1 (Fig. 4). Western blotting confirmed that vhs was not expressed in the Δvhs-infected cells, while other viral proteins VP16 and gD were expressed at similar levels to Wt virus (Fig. 4a). However, there was no difference in the timing or level of depletion of nectin1 from Δvhs- compared to Wt-infected cells, indicating that virus host shutoff was not responsible for nectin1 downregulation (Fig. 4b).

### Glycoprotein D is required for nectin1 downregulation in nTERT keratinocytes

**Fig. 5.**
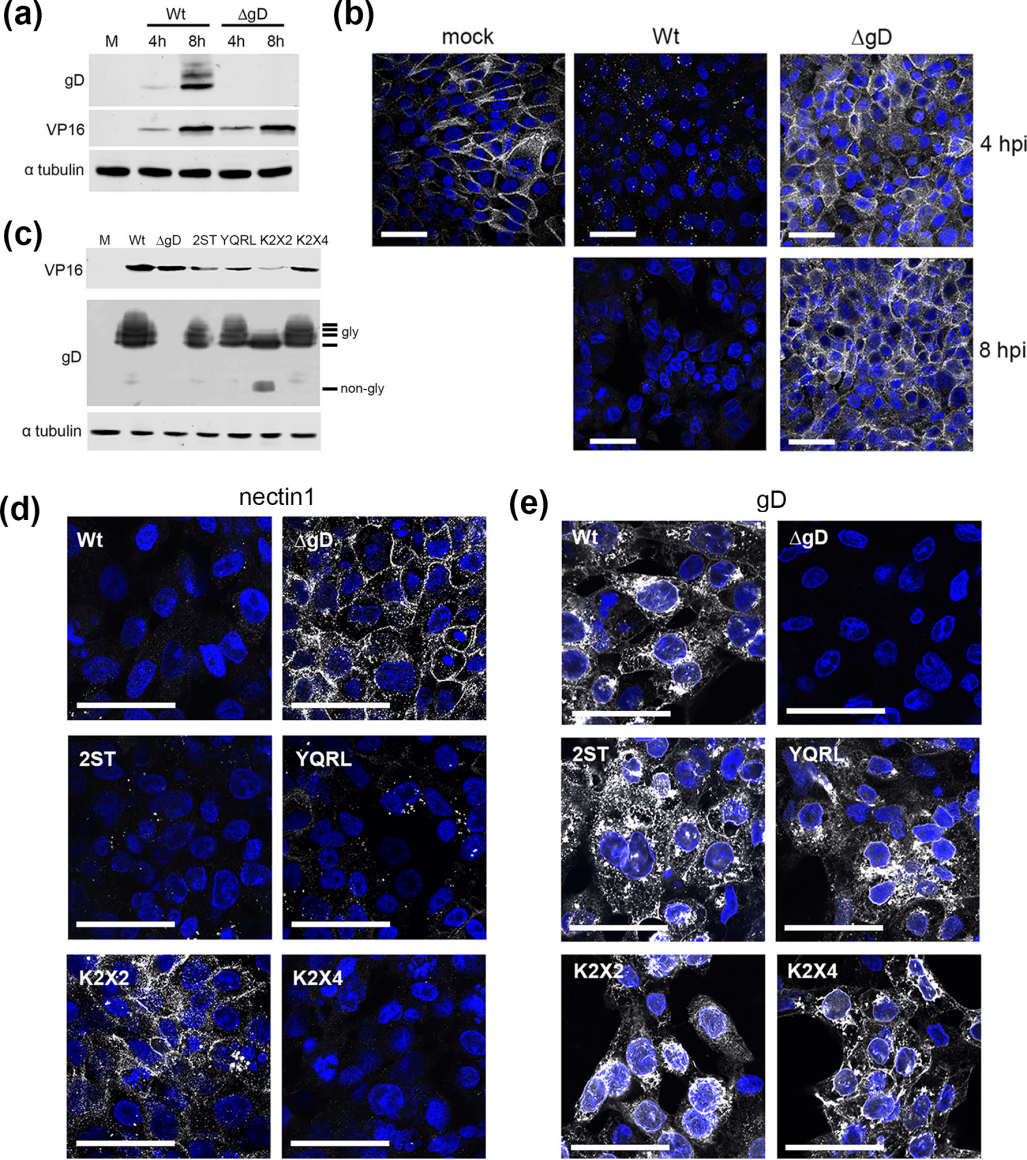
Downregulation of nectin1 in HSV1-infected cells requires gD. (**a**) nTERT cells grown on coverslips were infected with HSV1 Sc16 (Wt), or ΔgD at an m.o.i. of 2. Cells were fixed at 4 or 8 h and stained for nectin1 (white) and nuclei stained with DAPI (blue). Bar, 50 µm. (**b**) nTERT cells were infected with HSV1 Sc16 or HSV1 Sc16ΔgD at an m.o.i. of 5, lysates were harvested at the indicated times post-infection and subjected to SDS-PAGE and Western blotting for gD, VP16 and α-tubulin as a loading control. (**c**) nTERT cells grown on coverslips were infected with HSV1 engineered to express gD variants as indicated and harvested at 8 h post-infection and subjected to SDS-PAGE and Western blotting for gD, VP16 and α-tubulin as a loading control. (**d, e**) As for (**c**) but cells were grown on coverslips, fixed at 8 h post-infection and cell-surface stained for (**d**) nectin1 or (**e**) gD (white), and nuclei stained with DAPI (blue). Bar, 50 µm.

Given the difference in our results and those from others on the timing of nectin1 downregulation, we next investigated the role of gD in endogenous nectin1 downregulation using a deletion mutant for gD (ΔgD), which had been propagated on gD-expressing cells to incorporate gD into the virion to allow cell entry [29]. To confirm that viral protein production proceeded normally in ΔgD-infected nTERT cells, Wt- and HSV1 ΔgD-infected cells were harvested 4 and 8 hpi and subjected to SDS-PAGE and Western blotting (Fig. 5a). As expected, gD was present only in the Wt-infected cells – confirming that the ΔgD virus was of the expected phenotype – while VP16 expression was broadly similar in ΔgD and the parental Wt virus-infected cells, confirming that virus protein expression proceeded as normal during high multiplicity infection, even in the absence of gD (Fig. 5a). nTERT cells were then infected with Wt HSV1 or ΔgD and fixed and stained for cell surface nectin1 expression at 4 and 8 h (Fig. 5b). As before, in cells infected with Wt virus, nectin1 was clustered at the cell surface by 4 hpi and by 8 hpi it was no longer detectable. By contrast, in nTERT cells infected with the ΔgD virus, nectin1 remained evenly localized at the cell surface as late as 8 hpi (Fig. 5b, ΔgD). Hence, as nectin1 had not been downregulated during infection with the ΔgD virus which contains gD in the virions but does not express gD *de novo*, we conclude that newly synthesized gD is required for downregulation of nectin1 during HSV1 infection.

To further expand on the mechanism of gD downregulation of nectin1, we made use of a number of previously constructed viruses expressing a range of gD variants engineered to be retargeted to different compartments within the cell [29]: a KKXX motif (K2X2) where X is any amino acid, and which acts as an endoplasmic reticulum (ER) retention signal; a KKXXXX motif (K2X4) where the addition of the two additional residues overrides the ER retention signal [37]; a YQRL motif which retrieves protein from the plasma membrane to the endocytic pathway [38]; and a motif from the Golgi resident enzyme 2ST which acts a Golgi apparatus retention motif [39]. Of note, only HSV1 expressing the gD KKXX protein requires a gD complementing cell line for its propagation as all other variants were able to package gD for future rounds of infection [29]. Western blotting of infected cell extracts confirmed that all viruses expressed VP16 and gD (Fig. 5c). Moreover, all gD variants were similarly glycosylated, with the exception of the ER-retained variant which was present in the non-glycosylated and immaturely glycosylated forms (Fig. 5c). Cell surface staining of nectin1 and intracellular staining of gD in nTERT cells infected with each of these gD variant viruses revealed that only the ER-retained form of gD was unable to downregulate nectin1 from the plasma membrane (Fig. 5d, 5e K2X2). This suggests that gD does not act on nectin1 within the early secretory pathway and that nectin1 is targeted by gD in a post-ER compartment.

### Neither the ICP0 nor the Cbl E3 ubiquitin ligase is required for nectin1 downregulation in keratinocytes

**Fig. 6.**
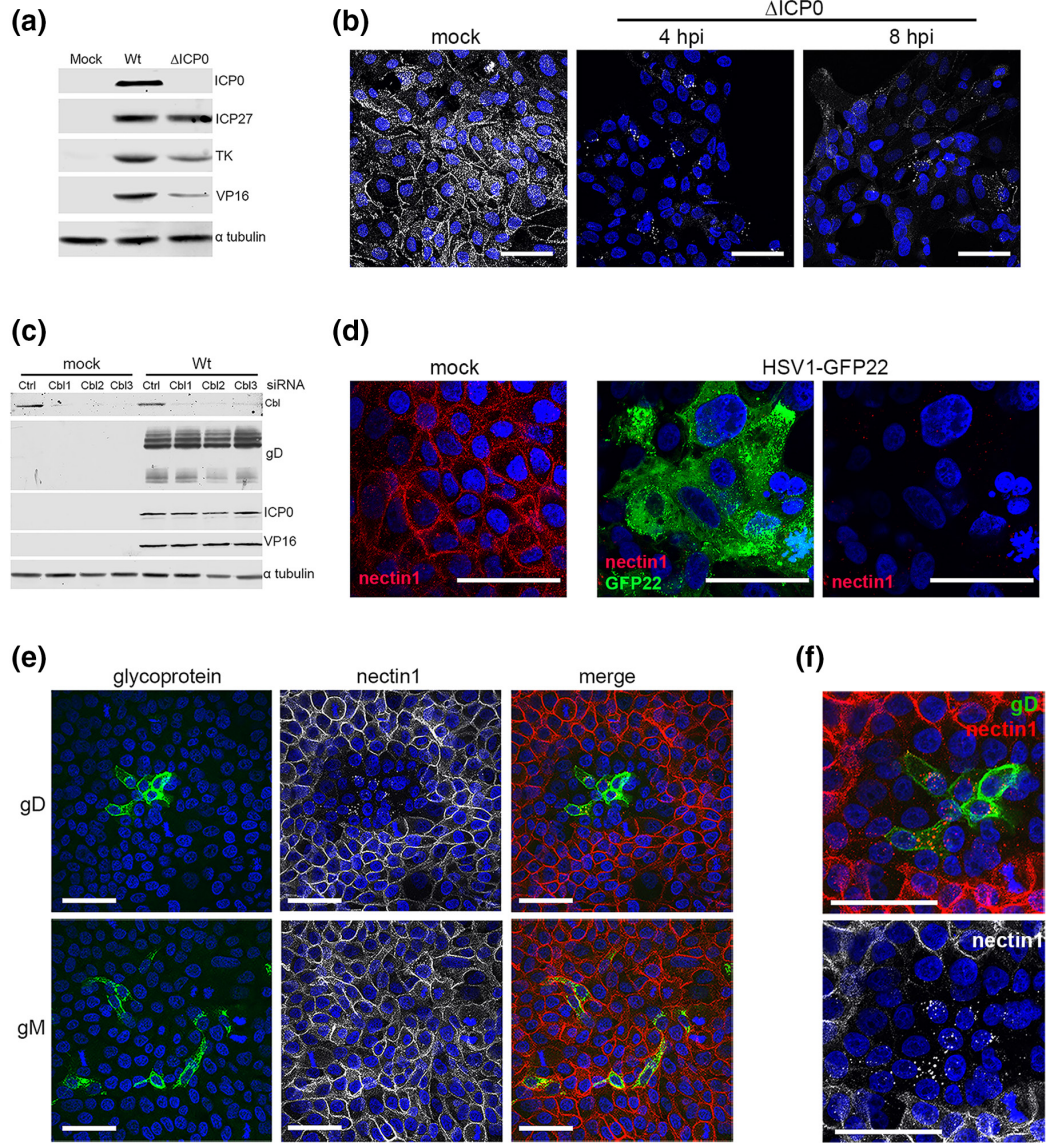
Downregulation of nectin1 in human keratinocytes does not require the virus-encoded E3 ligase ICP0, nor the cellular E3 ligase Cbl. (**a**) nTERT keratinocyte cells were infected with Wt (**s17**) or ΔICP0 virus at an m.o.i. of 2. Samples were harvested at 8 h and analysed by SDS-PAGE and Western blotting for the indicated proteins. (**b**) nTERT keratinocyte cells were infected with ΔICP0 virus at an m.o.i. of 2, fixed at 4 and 8 h, cell-surface staining was carried out for nectin1 (white) and nuclei were stained with DAPI. Bar, 50 µm. (**c**) nTERT cells were reverse transfected with control siRNA (Ctrl) or siRNA to Cbl (Cbl1, Cbl2 and Cbl3), and 48 h later were left uninfected or infected with Wt HSV1 at an m.o.i. of 5. After 8 h cells were harvested and analysed by SDS-PAGE and Western blotting for the indicated proteins. (**d**) nTERT cells were reverse transfected with Cbl3 siRNA and after 48 h were left uninfected (mock) or were infected with HSV1-GFP22. After 8 h cells were fixed and cell surface stained for nectin1 (red) and nuclei stained with DAPI (blue). Bar, 50 µm. (**e, f**) nTERT cells grown on coverslips were transfected with plasmid expressing HSV1 gD or gM. After 16 h cells were fixed, permeabilized and stained for nectin1 (red/white), and glycoprotein (green) and nuclei stained with DAPI (blue). Bar, 50 µm.

Previously published work has reported that both the HSV1-encoded E3 ubiquitin ligase ICP0 and the cellular E3 ligase Cbl are involved in nectin1 degradation [13] with both proteins found in a complex with gD during infection of Hep2 cells overexpressing nectin1. Hence, we next determined if either or both of the ubiquitin E3 ligases ICP0 or Cbl were required in keratinocytes. First, nTERT keratinocytes were infected with Wt or ΔICP0 HSV1, and protein production was compared between the two viruses at 8 h. Western blotting for a range of virus proteins of different kinetic classes indicated that the immediate early protein ICP27 was expressed at a similar level in ΔICP0- and Wt-infected cells, while as expected, the early protein TK and the late protein VP16 were expressed, but at a reduced level to that in Wt-infected cells (Fig. 6a). Nonetheless, cell surface staining of nTERT cells infected with ΔICP0 and fixed at 4 and 8 h after infection showed that nectin1 was efficiently downregulated in these cells, despite a lower level of protein expression, suggesting that ICP0 is not required for this activity in HSV1-infected keratinocytes (Fig. 6b).

To next determine if the cellular Cbl E3 ligase is involved in nectin1 downregulation, three different siRNA molecules were separately used to deplete the expression of Cbl in nTERT keratinocytes, and cells were left uninfected or were subsequently infected with Wt HSV1 for 8 h. Western blotting for Cbl confirmed efficient knockdown in both uninfected and HSV1-infected nTERT cells that had been transfected with each of the siRNAs (Fig. 6c, Cbl). Moreover, blotting for a number of virus proteins indicated that depletion of Cbl had no effect on the level of virus proteins produced during infection, including gD itself (Fig. 6c). Cell surface staining for nectin1 in Cbl-depleted cells confirmed that nectin1 was maintained at the cell surface of uninfected cells in the absence of this cellular E3 ligase (Fig. 6d, mock). Nonetheless, in Cbl-depleted cells which had been infected with HSV1-GFP22 and fixed at 8 h, nectin1 had been efficiently depleted from the cell surface (Fig. 6d, HSV1-GFP22). Taken together, these results suggest that in contrast to other work [13], neither ICP0 nor Cbl is involved in gD-induced depletion of endogenous nectin1 in human keratinocytes.

Previous studies have shown that gD alone is sufficient to downregulate overexpressed nectin1 from the cell surface of transiently expressing cells both *in cis* and *in trans* [9,11, 12]. To investigate if gD alone can downregulate nectin1 in the human keratinocyte system, cells were transfected with plasmid expressing either gD or a second HSV1 glycoprotein (gM) and stained 16 h later for nectin1 and either gD or gM (Fig. 6e). Expression of gD in isolation had a similar effect on nectin1 localization as virus infection, with cells which were expressing a high level of gD containing no, or very little, nectin1 at the cell surface (Fig. 6e). Staining of cells expressing gM showed that this second glycoprotein had no effect on nectin1 and confirmed that nectin1 depletion was specific to gD (Fig. 6e). Strikingly, nectin1 was also depleted from nTERT cells which did not express gD but were localized next to gD-positive cells suggesting that gD acts *in trans* as well as *in cis* (Fig. 6e), with a higher magnification image indicating that this effect may even extend to the next layer of cells away from the gD-expressing cells (Fig. 6f). This suggests that the expression of gD alone is sufficient to induce nectin1 depletion not only in gD-expressing cells but also in surrounding cells.

### HSV1 inhibits NK cell activation by nTERT keratinocytes in a gD-independent fashion

**Fig. 7.**
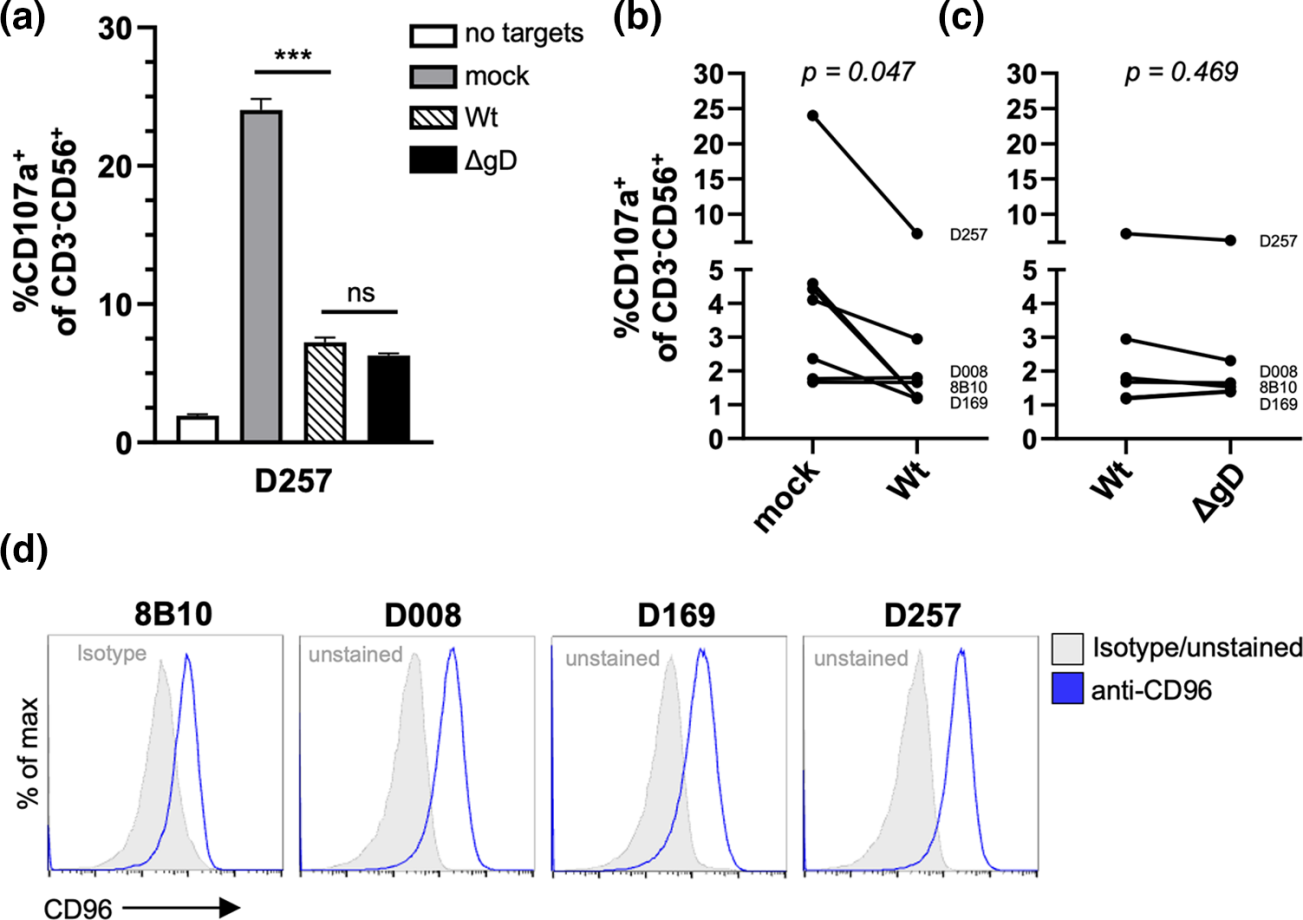
HSV1 inhibits NK cell activation in a gD-independent fashion. (**a**) Activation of an NK cell line (donor code shown) against nTERT cells, mock-infected, or infected with the indicated HSV1 strains. An effector:target ratio of 10 : 1 was used. Data are mean+sem of quadruplicate samples. Brown–Forsythe ANOVA with Dunnett’s T3 multiple comparison post-test showed significance at ***P<0.001. Summary activation data from seven NK cell lines against nTERT cells, (**b**) mock- or Wt-infected, and (**c**) Wt- and ∆gD-infected. Each data point represents the mean of quadruplicate or triplicate samples. Wilcoxon matched-pairs signed rank test showed significance as indicated. (**d**) Flow cytometric histogram overlays showing surface CD96 expression of indicated NK cell lines against isotype stained or unstained cells.

It has been reported recently that nectin1 interacts with the NK cell receptor CD96 and when overexpressed on human erythroleukaemia K562 cells leads to their increased susceptibility to NK cell cytotoxicity [14]. Our nTERT keratinocyte model therefore offered the opportunity to test if nectin1 acts as an NK cell ligand during HSV1 infection, since nectin1 is removed from the cell surface of Wt- but not ΔgD-infected cells (Fig. 5). Detached mock-infected, Wt- or ∆gD-infected nTERT cells were co-cultured with NK cell lines in the presence of an anti-CD107a antibody and degranulation (as a measure of activation) was measured by flow cytometry. This showed that keratinocytes were able to activate NK cells from a number of donors and that this activation was blocked by Wt HSV1 infection (Fig. 7a, b). Unexpectedly, there was no difference between NK cell activation against Wt HSV1-, compared to ∆gD-, infected cells (Fig. 7a, c), even though effector NK cell lines expressed the receptor for nectin1, CD96, on their surface (Fig. 7d). These observations are consistent with alterations in NK function during HSV infection being independent of gD and, by extrapolation, nectin1.

### gD-dependent nectin1 downregulation prevents HSV1 superinfection

**Fig. 8.**
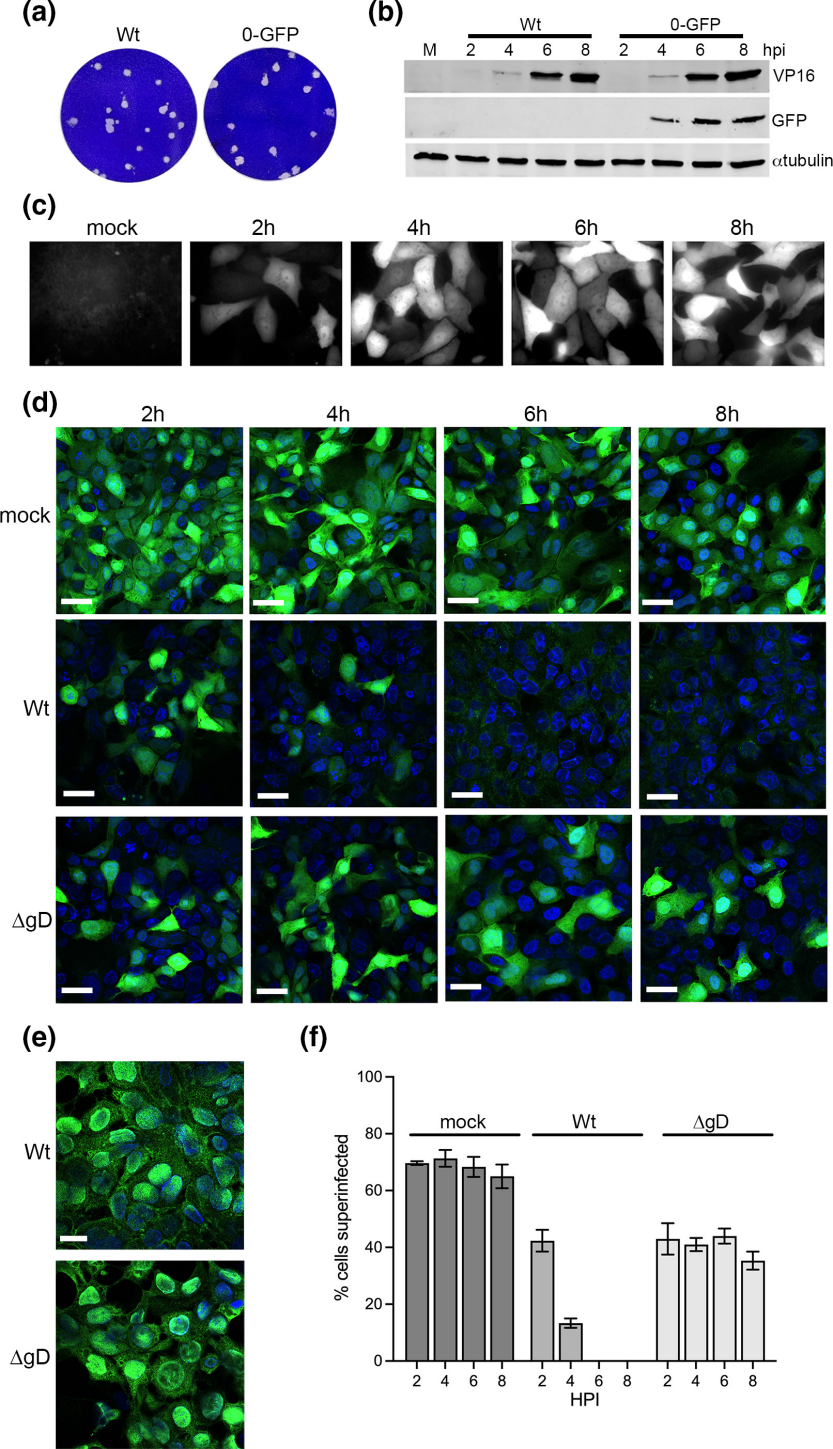
Superinfection exclusion of HSV1 infection is specific to gD expression. (**a**) Plaque size assay of Sc16 compared to Sc16 0-GFP which expresses GFP from the ICP0 promoter inserted into the UL26–UL27 intergenic region. (**b**) Vero cells were infected with Sc16 or Sc16 0-GFP, harvested at the indicated times, and subjected to SDS-PAGE and Western blotting for the indicated proteins. (**c**) Vero cells grown in a live-cell coverslip chamber were infected with Sc16 0-GFP at an m.o.i. of 5 and imaged for GFP fluorescence (white) at the indicated times. (**d**) nTERT cells grown on coverslips were either left uninfected (mock) or infected with Sc16 (Wt) or ΔgD viruses at an m.o.i. of 5. At the indicated times, these cells were infected with Sc16 0-GFP at an m.o.i. of 5 and all cells were fixed at 12 h, nuclei stained with DAPI (blue) and imaged using a Nikon A1 confocal microscope for GFP fluorescence (green). Bar, 20 µm. (**e**) Cells infected at T=0 with Sc16 (Wt) or ΔgD viruses at an m.o.i. of 5 were fixed at 12 h, permeablized and stained for the immediate-early protein ICP27 (green) to confirm efficient infection by both viruses. (**f**) Three biological replicates of the experiment described in (**d**) were carried out, and cells were counted for GFP fluorescence at each time point using ImageJ. HPI, hours post-infection that cells were superinfected.

An alternative reason for virus-induced cell surface receptor downregulation could be the exclusion of superinfection by newly infecting particles. Not only would this block infection once the first virion has entered, but could potentially prevent the misdirection of newly released virions to re-infect the cells they have just left. To test the role of nectin1 depletion in blocking superinfection of keratinocytes, we first constructed a virus that expresses GFP under control of the ICP0 promoter in the intergenic region between UL26 and UL27 of the Sc16 strain, the same Wt virus backbone as the ΔgD virus. This virus – named Sc16 0-GFP – produced plaques of an equivalent size as Sc16 (Fig. 8a) and expresses virus proteins with similar kinetics (Fig. 8b), and expressed GFP to levels detectable by 2 h (Fig. 8c). Hence this virus has a Wt phenotype but expressed GFP with immediate-early kinetics. To measure the ability of this virus to superinfect already infected nTERT keratinocytes over time, cells were first left uninfected (mock) or infected with Sc16 or ΔgD at an m.o.i. of 5. These cells were then infected with Sc16 0-GFP at an m.o.i. of 5 at the indicated times (Fig. 8d) and all were fixed at 12 h, stained with DAPI and imaged for GFP fluorescence (Fig. 8d). In cells that were originally uninfected, GFP fluorescence was present in the majority of cells throughout the time course (Fig. 8d, mock). By contrast, if cells had been infected with Sc16 at time 0 (T=0), some GFP was expressed if superinfection was carried out at 2 h, but these numbers were reduced at 4 h, and had disappeared by 6 h (Fig. 8d, Sc16). Intriguingly, when the same superinfection time course was carried out on cells infected with ΔgD at T=0, GFP was expressed throughout, indicating that superinfection was still possible in these cells as late as 8 h (Fig. 8d, ΔgD). To confirm that Wt and ΔgD viruses had infected nTERT cells efficiently, cells infected at the same time were left without subsequent superinfection, fixed at 12 h and stained for the immediate-early protein ICP27 (Fig. 8e). Three biological replicates of this experiment were performed with the number of GFP-expressing cells quantified in each sample, confirming the results shown in Fig. 8(d, f). These data indicate that the ability of HSV1 to exclude superinfecting virus is specific to the expression of gD, and given the timing of virus exclusion in Wt-infected cells, correlates with the gD-specific removal of nectin1 from the keratinocyte cell surface, suggesting that it is the removal of nectin1 by gD that is important.

## Discussion

In this study we have utilized the nTERT human keratinocyte system to investigate the outcome of HSV1 infection on its major entry receptor, nectin1, a member of the immunoglobulin superfamily that localizes to cell junctions and regulates cell adhesion, structural integrity, differentiation and communication networks [40]. Because we have been able to examine endogenous nectin1 in a cell type that HSV1 infects naturally in humans, our results not only offer a representation of events in HSV1 infection in its host but also help refine some of the discrepancies from previous work. Our results confirm that endogenous nectin1 is downregulated in HSV1-infected human keratinocytes, and reveal that this process does not involve the activity of the viral endoribonuclease vhs, a protein which targets the cellular transcriptome for degradation [41] and therefore has the potential to downregulate the cellular proteome. We further show that nectin1 downregulation requires newly synthesized gD rather than incoming gD on the virion, with nectin1 reorganization beginning around 2 h and depletion being complete by 6 h after infection in these cells. Moreover, gD was shown to be sufficient for downregulation of nectin1, indicating that no other virus proteins are necessary for the process.

Nectin1 is a type 1 transmembrane protein with three extracellular Ig-like domains, which forms *cis*- homodimers at the plasma membrane, and *trans*- homo- or heterodimers across cell junctions by interactions through the distal Ig V domain, thereby linking the actin cytoskeletons of two cells [42]. gD also binds to the V domain using the same residues that are involved in nectin1 dimerization and has the potential to disrupt nectin1 dimerization in *cis*- or *trans*- [43]. To date, there is little known about the turnover of nectin1 in cells, other than its role in forming adherens junctions [42,44], but the ability of gD alone to induce its depletion might suggest that destabilisation of nectin1 dimers by gD either accelerates its normal turnover pathway or activates a novel, virus-specific degradation pathway. Downregulation of nectin1 could occur in the early secretory pathway by ER-associated degradation (ERAD), where proteins are ubiquitinated at the ER, retrotranslocated into the cytosol and degraded by the proteasome before they have had a chance to reach the plasma membrane; this is exemplified by HIV1 Vpu which sequesters the HIV1 cell surface receptor CD4 in the ER and induces its degradation [6]. However, our results with viruses expressing differentially engineered gD variants, redirected to different subcellular compartments, suggest that gD is unable to alter nectin1 turnover when it is retained in the early secretory pathway, and therefore this activity is likely to occur at a post-ER compartment or more likely the cell surface. Further work is required to determine the fate of nectin1 and the involvement of various cellular protein turnover pathways in nectin1 depletion from the cell surface, in particular the nature of the nectin1 clusters that are formed at the cell surface prior to removal, but the fact that gD alone can induce nectin1 downregulation provides us with an excellent molecular model for unravelling this process. Moreover, further studies will allow us to determine how expression of gD in isolation causes the downregulation of nectin1 in cells surrounding gD-expressing cells and potentially in the cells next to those, which if proved, would suggest a paracrine effect of gD.

Recent work has shown that nectin1 can interact with the NK cell receptor CD96, and that overexpression of nectin1 increases cellular susceptibility to NK cell cytotoxicity [14], providing a rationale involving immune evasion for nectin1 downregulation by gD. Here, we found that nTERT keratinocytes were able to activate NK cells from a number of donors, but while HSV1 infection blocked this NK cell activation, there was no difference between the NK responses to Wt- or ΔgD-infected keratinocytes, despite the differential presence of nectin1 on the cell surface of these infected cells. As such, our data are consistent with nectin1 downregulation not being an immune evasion strategy used by HSV1. Control of NK responses by nectin1, however, is complex, involving multiple ligands (CD155/PVR, nectin1, nectin2) binding to several NK receptors capable of both activating and inhibitory signals (TIGIT, DNAM1 and CD96) [45,46]. How HSV1 infection alters these other ligands combined with the receptors expressed on the NK cells would probably influence the overall NK response triggered by CD96/nectin1 interactions. In addition, HSV1 infection has been shown to downregulate ligands for the activating NKG2D receptor, namely MICA, ULBP1, 2 and 3 [47,48], which may dominantly mask any potential effects of nectin1 downregulation on NK cell function. Regardless, the fact that NK cells recognize and kill HSV1-infected cells in the host [49] means that immune evasion would ultimately not be sufficient to completely overcome this host response.

While superinfection exclusion is a useful readout for gD-induced nectin1 downregulation, this mechanism could be interpreted as being more important for egressing virions than newly infecting virus. Nectin1 depletion occurs ahead of the release of progeny virions and, as such, the primary role for superinfection exclusion may be to prevent egressing progeny virions becoming trapped on the receptor on the plasma membrane as they leave the infected cell and/or to inhibit their re-entry into the infected cell. Some viruses overcome this issue by encoding their own factors that are incorporated into virions, aiding release from the cell surface after egress, for example the neuraminidases encoded by the influenza A viruses [50], but for many viruses the method of bypassing such entrapment is unknown. An additional consequence of nectin1 downregulation could be to block assembly of nectin1 into the HSV1 virion, an activity that has the potential to block subsequent binding of virion gD to downstream receptor molecules in a manner similar to that shown for CD4 incorporation into the HIV virion [1]. It is important to note that these potential reasons for nectin1 downregulation may not be mutually exclusive.

Further detailed molecular studies will be required to understand precisely how and why gD causes nectin1 depletion. Nonetheless, our work presented here has for the first time delineated the downregulation of nectin1 in HSV1-infected keratinocytes, the cell type that HSV1 infects in the human host, and has opened up new avenues of investigation that will enhance our understanding of the virus–host cell relationship.
